# Chronological Magnetic Resonance Angiography Demonstration of Spontaneous Posterior Cerebral Artery Recanalization: Unmasking Cardioembolic Stroke and Postprandial Atrial Fibrillation Mislabeled as Dumping Syndrome

**DOI:** 10.3390/diagnostics16213529

**Published:** 2026-10-27

**Authors:** Koji Hayashi, Hiroaki Maeda, Mamiko Sato, Asuka Suzuki, Yuka Nakaya, Hiromi Hayashi, Kouji Hayashi, Toyoaki Miura, Yasutaka Kobayashi

**Affiliations:** 1 Department of Rehabilitation Medicine, Fukui General Hospital, Fukui 910-8561, Japansatomoko@f-gh.jp (M.S.);; 2 Graduate School of Health Science, Fukui Health Science University, Fukui 910-3113, Japan; khayashi@fukui-hsu.ac.jp (K.H.); yasutaka_k@fukui-hsu.ac.jp (Y.K.)

**Keywords:** atrial fibrillation, ischemic stroke, dumping syndrome, gastrectomy, diagnostic errors, postprandial period

## Abstract

Atrial fibrillation (AF) and post-gastrectomy dumping syndrome share overlapping symptoms, such as palpitations, dizziness, and chest discomfort, making them clinically difficult to distinguish. We report a case where anchoring bias led both the patient and clinicians to misattribute postprandial symptoms to dumping syndrome, until a cardioembolic stroke unmasked latent AF. A 63-year-old man with a history of distal gastrectomy performed 5 years prior developed postprandial palpitations, chest discomfort, and dizziness triggered by rapid eating or cold drinks. A prior facility had empirically attributed these episodes to dumping syndrome without cardiac evaluation. Initial electrocardiogram showed normal sinus rhythm. The next day, he developed acute diplopia and ptosis due to left oculomotor nerve palsy. Brain MRI demonstrated a left medial midbrain infarction, and MR angiography (MRA) showed 99% stenosis of the left P1 segment. Transesophageal echocardiography demonstrated left atrial enlargement and decreased left atrial appendage flow velocity with spontaneous echo contrast. Continuous electrocardiographic telemetry post-dinner captured paroxysmal AF, unmasking the arrhythmia. His CHA_2_DS_2_-VASc and HAS-BLED scores were calculated as 3 and 2, respectively. He was treated with anticoagulants and discharged with mild sequelae (modified Rankin Scale score 1). Follow-up MRA at 3 months showed complete recanalization of the P1 segment. This chronological MRA sequence provided visual proof differentiating cardioembolism from fixed intracranial atherosclerosis.

**Figure 1:**
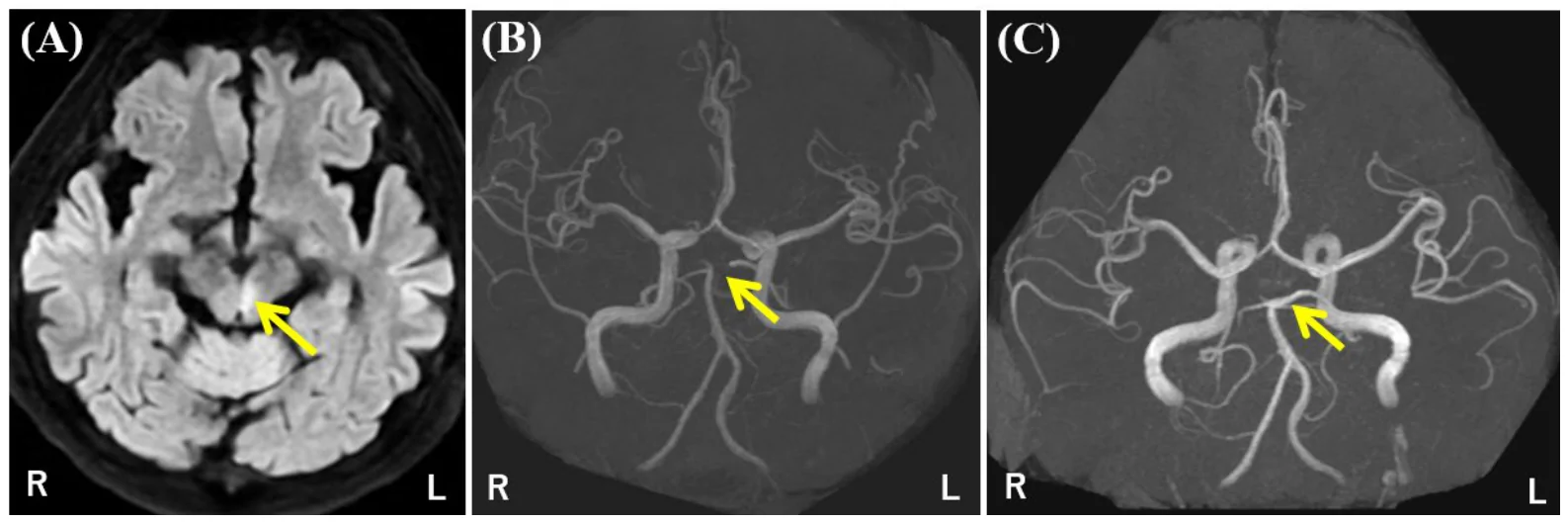
Chronological brain magnetic resonance imaging (MRI) and magnetic resonance angiography (MRA) findings providing etiological proof of cardioembolic stroke. (**A**) Diffusion-weighted brain MRI on admission reveals a focal hyperintensity in the periaqueductal region near the left oculomotor nucleus in the midbrain (arrow). (**B**) MRA on admission shows severe signal loss (99% stenosis/disconnection) in the P1 segment of the left posterior cerebral artery (arrow), with distal vessel flow preserved. (**C**) Follow-up MRA three months after discharge demonstrates complete, spontaneous recanalization of the left P1 segment without any residual vessel wall stenosis or irregularity (arrow). R: right, L: left. While an isolated initial MRA showing a severe P1 segment lesion (Figure 1B) cannot reliably differentiate fixed intracranial atherosclerosis (ICAD) from an embolic occlusion, the present serial MRA sequence provides critical diagnostic proof. The complete, spontaneous resolution of the 99% P1 occlusion on follow-up MRA (Figure 1C) without residual arterial wall stenosis visually confirms dynamic thrombus dissolution. Combined with the absence of significant carotid intima-media thickening on ultrasonography and the presence of left atrial enlargement (51 mm), decreased left atrial appendage (LAA) flow velocity (28 cm/s), and spontaneous echo contrast (SEC) on transesophageal echocardiography (TEE), this chronological image set provides strong evidence supporting a cardioembolic mechanism (AF embolism) rather than primary atherothrombosis [1].

**Figure 2:**
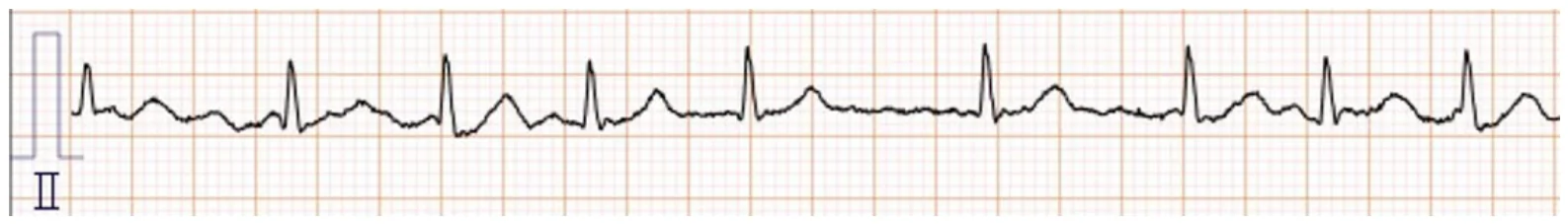
Lead II excerpt from a 12-lead electrocardiogram (ECG) recorded immediately following continuous telemetry monitor alarm after dinner on Day 1. The calibration signal (10 mm/mV, 25 mm/s) is shown in grey, while the lead label “II” and the ECG waveform are shown in black. On admission (Day 1), the patient experienced precordial discomfort identical to his previously attributed “dumping syndrome” symptoms immediately after dinner. When the continuous telemetry monitor detected a rhythm abnormality, a standard 12-lead ECG was performed immediately, capturing paroxysmal atrial fibrillation (AF) during the acute symptomatic episode. Figure 2 displays the Lead II excerpt from this 12-lead ECG, providing definitive objective evidence of the meal-triggered arrhythmia. Treatment was initiated with intravenous heparin and edaravone immediately after admission, transitioning to oral edoxaban (60 mg/day) on Day 2. Sinus rhythm was spontaneously restored on Day 3. The patient was discharged on Day 40 with mild residual diplopia (modified Rankin Scale score 1). At the 4-month follow-up, he underwent catheter ablation (pulmonary vein isolation) for recurrent paroxysmal AF, and no recurrent ischemic events occurred. This case exemplifies anchoring bias, wherein a cardioembolic stroke ultimately unmasked AF that had been masquerading as dumping syndrome. AF can also present with palpitations, tachycardia, dizziness, and chest discomfort, closely mimicking dumping syndrome. Furthermore, the risk of AF increases following gastrointestinal surgery and as gastric cancer advances in stage [2,3]. Triggers for postprandial AF include swallowing, ingestion of ice-cold liquids, and alcohol consumption [4,5,6]. The underlying pathophysiological mechanisms involve elevated vagal tone during or after meals—leading to vagally mediated AF—as well as direct mechanical stimulation of the posterior left atrium caused by esophageal distention from a food bolus [4,5,6]. Additionally, deglutition can trigger a vagal reflex that shortens atrial refractoriness in a non-uniform manner, resulting in dispersion and micro-reentry circuits [4,5]. Esophageal stimulation may also evoke an adrenergic reflex, leading to asynchronous atrial depolarization and focal reentry [4,5]. Ingestion of ice-cold water may provoke sympathetic surge atop high vagal tone, creating an autonomic conflict recognized as a potent trigger for arrhythmias [4]. Consequently, AF can occur immediately after meals, highlighting the necessity of considering arrhythmia in the differential diagnosis of postprandial distress. In this patient, AF was masked by postprandial symptoms that were empirically misattributed to dumping syndrome until a cardioembolic stroke occurred. Following the initial diagnosis of dumping syndrome, both the patient and healthcare providers fell victim to anchoring bias, attributing all subsequent postprandial symptoms to the gastrointestinal diagnosis without pursuing cardiac evaluation [7]. While hindsight bias must be acknowledged when evaluating these clinical decisions retrospectively [8]—recognizing that postprandial distress after gastrectomy reasonably suggests dumping syndrome initially—clinicians must avoid prematurely anchoring on prior diagnoses without objective verification. Given that AF can be triggered by deglutition or food intake, clinicians should maintain a high index of suspicion and perform prompt pulse or ECG monitoring when cardiovascular symptoms occur postprandially. This case serves as a critical educational reminder regarding the diagnostic pitfalls associated with cognitive biases, particularly anchoring and hindsight biases [7,8]. **Study Limitations:** One specific limitation of this case report should be noted. The prior diagnosis of dumping syndrome, established at an outside facility, was purely empirical and lacked formal objective confirmation, such as oral glucose tolerance testing, gastric emptying scintigraphy, or validated symptom scoring. Although this empirical attribution directly illustrates the real-world clinical pitfall of anchoring bias in post-gastrectomy patients, objective diagnostic verification of the gastrointestinal disorder was not performed prior to the stroke presentation.

## Data Availability

The data presented in this study are available on request from the corresponding author. Due to patient privacy and ethical considerations, the data are not publicly accessible.
